# Poster Session I - A130 A COHORT REVIEW OF CELIAC PATIENTS WITH NORMAL SEROLOGY. IS FOLLOW-UP EGD INDICATED?

**DOI:** 10.1093/jcag/gwaf042.130

**Published:** 2026-02-13

**Authors:** B D Blake, C Medvedic

**Affiliations:** Gastroenterology, University of Manitoba Max Rady College of Medicine, Winnipeg, MB, Canada; Gastroenterology, University of Manitoba Max Rady College of Medicine, Winnipeg, MB, Canada

## Abstract

**Background:**

The role of follow-up small intestinal biopsy in celiac disease is unclear. Current guidelines do not recommend follow-up duodenal biopsies to confirm histologic recovery. The reported sensitivity and specificity of IgA TTG antibody (TTG) to predict histologic recovery in patients on a gluten free diet (GFD) is 50% and 83% respectively. Therefore, it is possible that patients may have ongoing villous injury despite normalization of their TTG. A frequent practice is to biopsy the small intestine of celiac patients undergoing endoscopy for other reasons.

**Aims:**

The aim of this descriptive retrospective study is to evaluate the role of small intestinal biopsy when investigating non celiac related symptoms in patients with normalization of TTG.

**Methods:**

Adult patients with non-celiac symptoms from the Celiac Clinic at St Boniface Hospital between January 2020 to April 2025 who had normalization of celiac serologies and follow-up gastroscopy with small intestinal biopsy were included. Patient demographics, laboratory investigations, and pathology was collected.

**Results:**

Of 477 patients in the celiac clinic cohort of 477 patients, 47 met inclusion criteria. Mean age was 47 with 71% being female. Mean follow-up labs included hemoglobin (140g/L), ferritin (120ug/L), Vitamin B12 (485pmol/L), TSH (2.1mIU/L), AST (22U/L), ALT (22U/L), Vitamin D (90nmol/L). 45% of patients had a family history of celiac disease and 100% had self-reported good adherence to a GFD. 62% were symptomatic to gluten exposure. Associated diseases included type 1 diabetes (2%), thyroid disease (21%), and microscopic colitis (7%). Mean baseline and follow-up TTG (normal < 19U/mL) at the time of endoscopy was 166.2 and 9.1, respectively. Indications for gastroscopy included dyspepsia, refractory reflux, and imaging abnormalities. All follow-up gastroscopies (N = 54) noted normal endoscopic duodenal mucosa. Follow-up small intestinal pathology was reported separately as duodenal bulb and second part duodenum (D2) in 37% of samples. Follow-up Marsh (M) histology was as follows: 0 (74%), 1 (17%), 2 (1.9%), 3a (3.7%), 3b (1.9%), 3c (1.9%). 91% of patients had M0 or 1 and 9% of patients had M2 or higher. Discordance was noted in 4/20 (20%) of samples: 2 had M3a in the bulb and M0 in D2, 1 had M2 in the bulb and M1 in D2 and 1 had M3c in the bulb and M3b in D2.

**Conclusions:**

In this cohort of celiac patients with normal TTG and good self-reported adherence to GFD, 91% of patients had normal (M0/1) follow-up small intestinal biopsies. Although few, a significant minority of patients had discordance between the histology of D2 and the bulb. The results suggest that follow-up gastroscopy and small intestinal biopsy is not routinely needed to confirm histologic recovery in patients with a normal TTG. Further study is warranted in a larger clinic cohort including biopsies from D2 and the duodenal bulb.

**Funding Agencies:**

None

